# The effectiveness of an additive informal social network intervention for forensic psychiatric outpatients: results of a randomized controlled trial

**DOI:** 10.3389/fpsyt.2023.1129492

**Published:** 2023-05-24

**Authors:** Lise T. A. Swinkels, Thimo M. van der Pol, Jos Twisk, Janna F. ter Harmsel, Jack J. M. Dekker, Arne Popma

**Affiliations:** ^1^Department of Forensic Outpatient Care, Inforsa Forensic Mental Healthcare, Amsterdam, Netherlands; ^2^Department of Child and Adolescent Psychiatry and Psychosocial Care, Amsterdam Public Health Research Institute, Amsterdam UMC, VU University Amsterdam, Amsterdam, Netherlands; ^3^Department of Research and Quality of Care, Arkin Mental Health Institute, Amsterdam, Netherlands; ^4^Department of Epidemiology and Data Science, Amsterdam UMC, VU University Amsterdam, Amsterdam, Netherlands; ^5^Department of Clinical Psychology, VU University Amsterdam, Amsterdam, Netherlands

**Keywords:** forensic psychiatry, informal social network intervention, volunteer mentoring and befriending, mental wellbeing, psychiatric functioning, criminal recidivism, social support, randomized controlled trial

## Abstract

**Objectives:**

A supportive social network is associated with better mental health and wellbeing, and less criminal behavior. Therefore, this study examined the effectiveness of an additive informal social network intervention to treatment as usual (TAU) among forensic psychiatric outpatients.

**Materials and methods:**

An randomized controlled trial (RCT) was conducted in forensic psychiatric care, allocating eligible outpatients (*N* = 102) to TAU with an additive informal social network intervention or TAU alone. Participants receiving the additive intervention were matched to a trained community volunteer over 12 months. TAU consisted of forensic care (e.g., cognitive behavioral therapy and/or forensic flexible assertive community treatment). Follow-up assessments were conducted at 3, 6, 9, 12, and 18 months after baseline. The primary outcome was the between-group effect on mental wellbeing at 12 months. Between-group effects on secondary outcomes (e.g., general psychiatric functioning, hospitalization, criminal behavior) were explored.

**Results:**

Intention-to-treat analyses showed non-significant between-group effects on mental wellbeing on average over time and at 12 months. However, significant between-group effects were found on hospitalization duration and criminal behavior. Specifically, TAU participants were hospitalized 2.1 times more days within 12 months and 4.1 more days within 18 months than participants in the additive intervention. Furthermore, TAU participants reported 2.9 times more criminal behaviors on average over time. There were no significant effects on other outcomes. Exploratory analyses revealed that sex, comorbidity, and substance use disorders moderated effects.

**Conclusion:**

This is the first RCT examining the effectiveness of an additive informal social network intervention in forensic psychiatric outpatients. Although no improvements were found on mental wellbeing, the additive intervention was effective in reducing hospitalization and criminal behavior. The findings suggest that forensic outpatient treatment can be optimized by collaborating with informal care initiatives aimed at improving social networks within the community. Future research is warranted to determine which specific patients might benefit from the intervention and if effects can be improved by extending the intervention duration and enhancing patient compliance.

**Clinical Trial Registration:** [https://trialsearch.who.int/Trial2.aspx?TrialID=NTR7163], identifier [NTR7163].

## Introduction

Forensic psychiatric patients often face multiple and persistent mental problems as well as social network-related problems leading to criminal behavior or relapse into crime (1–3). An informal social network, consisting of connections with family, friends, peers, and romantic partners, is described as one of the values and “primary human goods” to be attained in forensic mental healthcare (4–6). Although two dominant models used to frame forensic mental healthcare – the Risk Need Responsivity (RNR) model and Good Lives Model (GLM) – emphasize the importance of social networks, to date, evidence-based social network interventions for forensic psychiatric patients have yet to be established (4, 7–9). Moreover, there is an ongoing need to further improve treatment effectiveness for this vulnerable population with complex needs (10–13).

A supportive social network is considered an important protective factor against mental health problems and criminal recidivism (14–18). Multiple cross-sectional studies found that social support is positively related to mental health (recovery) and mental wellbeing in general and psychiatric populations (19–22). Furthermore, qualitative studies showed that having a supportive, trustful, stable, and prosocial network positively influenced forensic patients’ perceptions of rehabilitation from institutions to society (23, 24). More specific, supportive relationships with both professionals and informal social networks were described as core factors that contributed to recovery both in general and forensic psychiatric care (24, 25). Although the relationship between social network factors and criminal recidivism is not fully understood (26), several studies did find that higher family support and social connections are related to lower criminal recidivism in forensic populations (27–31). Despite the promise of social network interventions as a means of improving treatment outcomes, enhancement of social networks outside the therapeutic environment is not often addressed in (forensic) psychiatric treatment (16, 32).

A variety of types of social network interventions have been distinguished for psychiatric populations, for example: social support groups, mutual help groups, and trained volunteers (32). Recent meta-analytic studies including different social network interventions showed small-to-negligible effects of these interventions in general on social support, and small-to-moderate effects on treatment outcomes (33, Swinkels, Hoeve et al. submitted). Although personalized interventions targeting patients’ social network and needs outside of the mental healthcare institute appear to be preferred over generic approaches, the most effective approach has yet to be determined (33). In addition, studies examining the effectiveness of social network interventions on treatment outcomes in forensic psychiatric populations are scarce (34). One small pilot randomized controlled trial (RCT) comparing a social support intervention to standard care among former prisoners with substance use disorders showed no between-group effects on social support, substance use, and criminal recidivism (35). A larger study examining a combined social network intervention, including peer support and group counseling, showed reduced alcohol use in forensic psychiatric patients receiving the additive combined intervention, compared to patients receiving standard care alone (36). The effectiveness of specific types and components (i.e., one-to-one provision of support or group counseling) of the combined social network intervention on treatment outcomes in forensic populations remains unclear. The results point to the need to further explore which types of social network interventions are effective in improving social networks and other relevant treatment outcomes for forensic populations.

The addition of a specific type of social network intervention using trained volunteers from the community (i.e., informal social network intervention) could be a promising approach, since forensic patients often rely on support from professionals (i.e., formal social networks) and are more likely to have network members with risky and criminal behaviors, or to lose support from friends and peers (26, 37). Over the past century these interventions, known as befriending (i.e., one-to-one contact between volunteers and participants focused on developing a supportive relationship) and mentoring interventions (i.e., one-to-one contact between volunteers and participants focused on achieving goals), have been applied and studied in various patient populations (38–40). Previous meta-analyses showed that (additive) befriending, compared to treatment as usual (TAU) or other control groups, modestly improved depressive symptoms and self-reported outcomes (i.e., mental health, wellbeing, and social network) in patient populations (38, 39). However, clinical trials on befriending among psychiatric patients are scarce (41, 42). In addition, to our knowledge, there are no studies examining the effects of befriending on treatment outcomes in forensic psychiatric patients.

Mentoring, on the other hand, has extensively been studied in forensic populations, such as youth at risk for delinquency, and to a lesser extent in adults returning to society from prison. Two meta-analyses examining young adolescent populations revealed modest effects of mentoring interventions on drug use, delinquency, and aggression among youth at risk for delinquency, as well as moderate effects on a range of psychosocial outcomes (43, 44). For adult forensic populations, two studies examining the effectiveness of mentoring interventions found reduced criminal recidivism in reentering offenders receiving the intervention compared to controls (45–47). Nevertheless, researchers emphasize that the evidence is still limited due to the variety of existing mentoring approaches examined and a limited description of the implementation, making it difficult to distinguish between effects (43, 44, 48). Moreover, the effectiveness of mentoring interventions on treatment outcomes in forensic psychiatric patients remains unclear, as previous studies have only examined treatment effects in at-risk youth and (sex) offenders, including participants with and without psychiatric disorders. Therefore, more rigorous studies with clearly defined informal social network interventions are warranted to explore whether a forensic psychiatric population might profit from these interventions.

Several reviews showed that informal social network interventions focused on developing a supportive relationship between volunteers and participants (i.e., befriending components), as well as goal-oriented and time-restricted approaches (i.e., mentoring components) improved effectiveness (43, 44, 48). Against this background, we adopted an informal social network intervention based on specific components of befriending and mentoring, entitled *Forensic Network Coaching* (FNC). FNC was provided by trained volunteer coaches from an informal care institute with longstanding experience in volunteer interventions for psychiatric populations. The primary aim of FNC was to establish a supportive non-professional relationship between volunteer coaches and participants receiving treatment at a forensic outpatient care institute. In addition, participant-coach dyads were stimulated to focus on social network-related goals: enhancing (1) the social networks (i.e., network size and the quality of social relationships), (2) social support, and (3) social participation. In order to promote compliance, we intended to provide an accessible informal social network intervention by tailoring social network-related goals to the needs of participants. Furthermore, an RCT design was used comparing the effectiveness of TAU with the addition of FNC to TAU alone, to clearly distinguish the benefits of the informal social network intervention. To our knowledge, this is the first RCT to examine the effectiveness of an additive informal social network intervention among forensic psychiatric outpatients, therefore, treatment effects of multiple outcome domains were explored.

The aim of this RCT was to examine whether the addition of FNC to TAU was more effective in improving mental wellbeing (i.e., primary outcome) compared to TAU alone. Furthermore, we explored the effectiveness of FNC plus TAU compared to TAU alone on general psychiatric functioning, hospitalization, criminal behavior, and incarceration (i.e., key secondary outcomes), as well as on other secondary treatment outcomes (i.e., social network, substance use, quality of life, and self-sufficiency). In addition, as engagement to the intervention was expected to vary between patients, we explored the treatment effects of primary and key secondary outcomes across patients with different levels of compliance to the FNC intervention (i.e., no, low, and high compliance). Lastly, potential moderators of treatment effects of primary and key secondary outcomes were explored to optimize knowledge regarding personalized social network interventions.

## Materials and methods

### Study design

This study, a mono-center open label RCT with two parallel groups, was conducted at Inforsa, the forensic outpatient care department of Arkin Mental Healthcare in Amsterdam, the Netherlands, between April 2018 and December 2022. All participants were recruited at three sites of Inforsa: (1) forensic flexible assertive community treatment (forensic FACT) teams for adults, (2) a forensic FACT team for (young) adolescents, and (3) a forensic outpatient clinic. The main goal of these forensic outpatient treatment teams is twofold: (1) reducing patients’ risk of criminal recidivism, contributing to enhanced safety in the society, and (2) enhancing patients’ mental health and wellbeing. We compared the effectiveness of treatment as usual with an additive informal social network intervention – *Forensic Network Coaching* (FNC) – to treatment as usual (TAU) alone. After screening for eligibility, participants were allocated to one of the two intervention groups using simple 1:1 randomization with random varied block lengths (i.e., 4 and 6), stratified by forensic outpatient care site. The randomization tool in Castor EDC, an online platform for clinical trials, was used for this procedure (49). Follow-up assessments took place at 3, 6, 9, 12, and 18 months after baseline assessment. Written informed consent was obtained from participants prior to baseline assessment.

More details about the RCT can be found in our study protocol, which was prepared according to the SPIRIT 2013 statement (50, 51). The Medical Ethics Committee of the VU University Medical Center (NL60308.029.17) reviewed and approved the RCT prior to data collection. The study is registered at the Netherlands Trial Register (NTR7163). The CONSORT 2010 Checklist was used to report this study (52, Supplementary material).

### Participants

#### Sample size

The power calculation for a repeated measured design, conducted in Stata (53), showed that a total of 68 participants were required to detect a small-to-medium effect size (Cohen’s *F* = 0.20) in a pairwise comparison of pre-post change between two active treatment arms, with a power of 0.80 and a within-person correlation coefficient of 0.50. We aimed to include 105 participants to account for 30% expected dropout from the intervention.

#### Inclusion and exclusion criteria

Participant were forensic psychiatric outpatients receiving treatment at Inforsa Forensic Outpatient Care. Patients were included if they (1) received at least 3 months in treatment and could adhere to appointments with their clinician, (2) were aged 16 years or older, (3) were diagnosed with a psychiatric diagnosis by a clinician according to the DSM-IV-TR/5, (4) had limitations in social network and social participation identified by their clinician and a research assistant using the Self-Sufficiency Matrix (54), and (5) were not completely satisfied with their social relationships, as defined with a score of five or below on one item of the Manchester Short Assessment of Quality of Life measuring the self-reported quality of social relationships on a 7-point Likert scale (55).

Patients were excluded if, before or at baseline assessment, (1) acute psychotic symptoms, (2) acute suicidality, (3) severe addiction problems requiring immediate intervention, and (4) a high risk for severe aggression toward clinicians or others were identified. Finally, patients could not participate if they were enrolled in other scientific research projects at Inforsa.

#### Study sample

**Figure 1:**
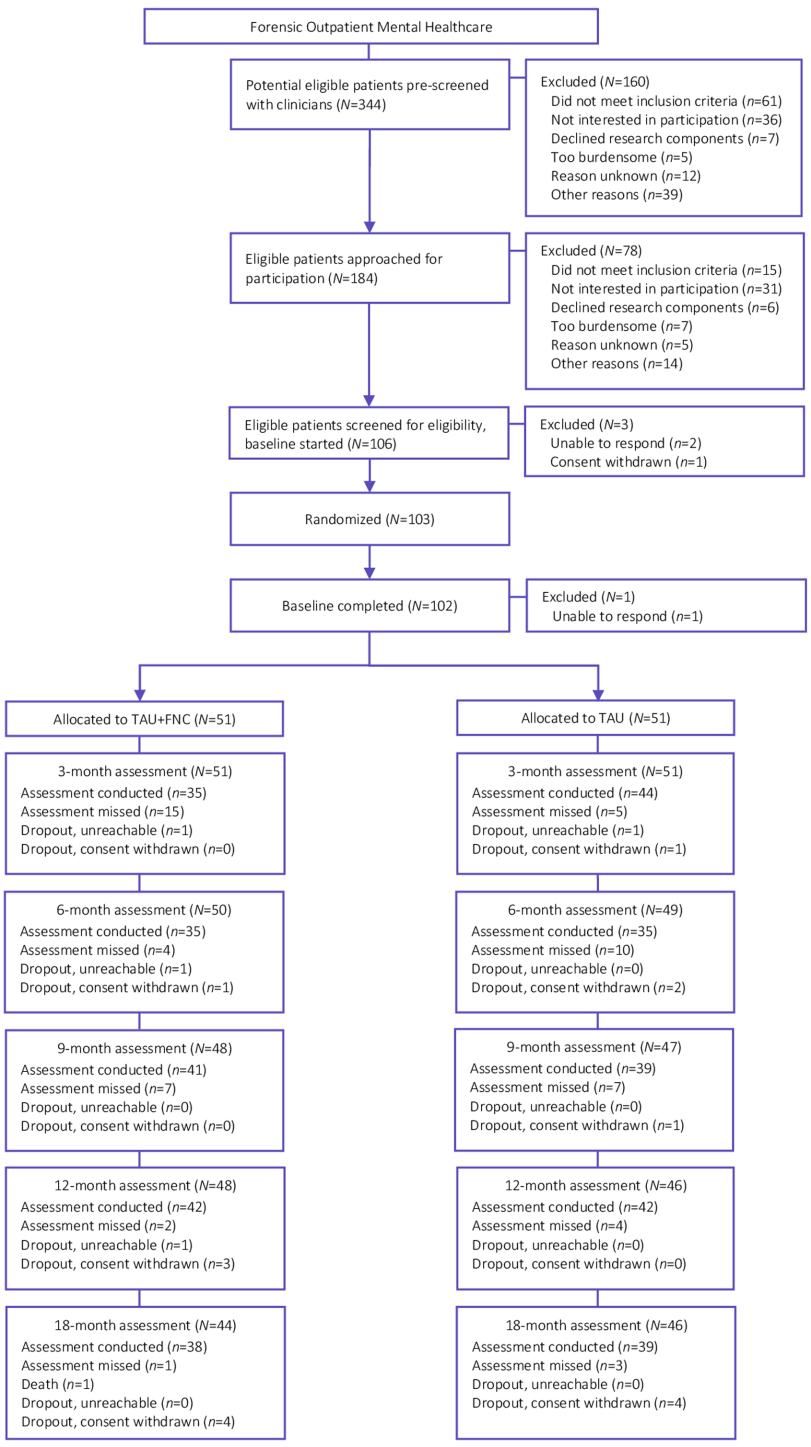
Trial flow diagram.

The flow diagram including a complete overview of the selection and assessment of participants in this trial is presented in Figure 1. A total of 184 potential participants were approached for eligibility after pre-screening, of which 106 participants were screened by research assistants to determine eligibility at baseline assessment. Subsequently, four participants were excluded before randomization, and one patient after randomization, because they were unable to respond (*n* = 3) or withdrew consent (*n* = 1). A remaining sample of 102 participants randomly assigned to TAU+FNC (*n* = 51) or TAU (*n* = 51) were included in the intention-to-treat sample.

**Figure 2:**
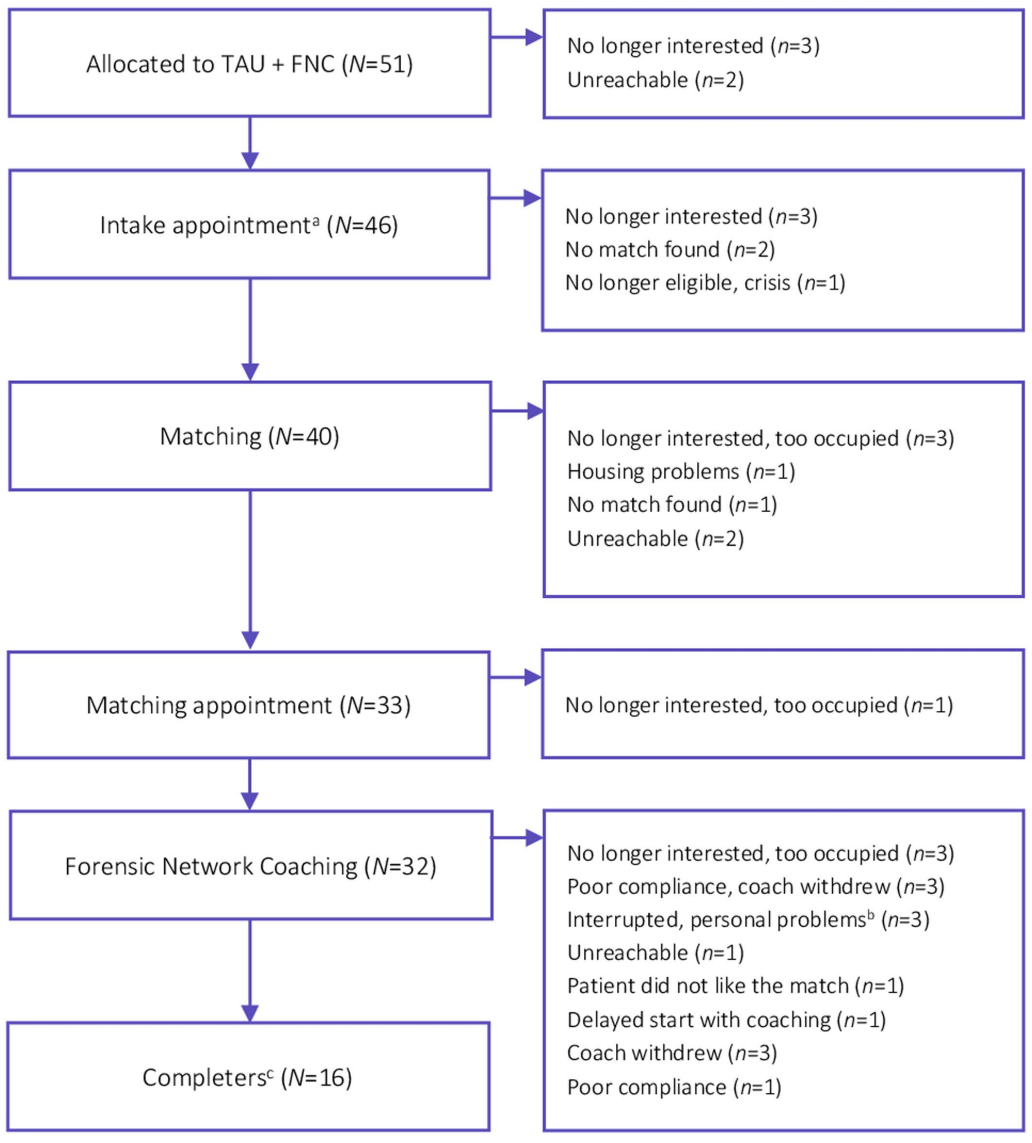
Forensic network coaching flow diagram. ^a^Organized by De Regenboog Groep [The Rainbow Group], an informal care service in Amsterdam. ^b^Both patient- and coach-related personal problems. ^c^Coaches and patients who are matched at least 10 months between baseline and 18-months follow-up assessment.

Notably, the amount of dropout in the FNC intervention intention-to-treat sample was higher than expected. A substantial group of participants failed to start with the FNC intervention (37%; *n* = 19) and in another group of participants (31%; *n* = 16) the intervention was prematurely terminated, as shown the Forensic Network Coaching trial diagram in Figure 2. Additional per-protocol samples were defined based on the *a priori* set minimal 10-month duration of the FNC intervention between baseline and 12-month follow-up. However, it was not always feasible to match participants to coaches within 2 months after baseline. Moreover, some patient-coach dyads continued the contact after the 12-month follow-up. Therefore, different per-protocol groups were defined based on the duration of the patient-coach contact from baseline to 18-month follow-up: (1) participants matched at least 10 months were included in the *high compliance* group (*n* = 16), (2) participants matched less than 10 months were included in the *low compliance* group (*n* = 16), and (3) participants who could or did not want to be matched to a coach were included in the *no compliance* group (*n* = 19).

### Interventions

#### Treatment as usual

Participants allocated to both groups received standard treatment at the outpatient care sites, which consisted of a variety of specific interventions, such as ambulatory psychotherapies (e.g., cognitive behavioral therapy, eye movement desensitization and reprocessing, pharmacotherapy), and/or forensic FACT (56). Additionally, clinicians of all participants included in our trial received information about a general and brief social network intervention on how to address social network enhancement in treatment. No treatments were withheld from participants. TAU could have been discontinued or terminated by clinicians and/or by participants during the study.

#### Forensic network coaching

Participants allocated to the TAU+FNC group were stimulated by researchers and/or clinicians to engage in volunteer coaching to improve the quality of social networks, social support, and social participation in addition to TAU, during 12 months after baseline assessment. The FNC intervention was provided by De Regenboog Groep [The Rainbow Group], an external informal care institute providing volunteer services for people with social or mental challenges who are lonely and/or have a psychiatric and/or an addiction background. De Regenboog Groep provided the selection, training, matching, and supervision of volunteer coaches – *Forensic Network Coaches*. Before the start of FNC, an intake appointment with the coordinator and patient took place to determine participants’ willingness to meet a coach, patient-specific network goals, and preferences. Participants who were unable or unwilling to participate in the intervention, were given the opportunity to start at a later timepoint, up to 12 months after baseline. Participants and coaches being matched were encouraged to meet with each other for a couple of hours, once every 14 days. However, patient-coach dyads were free to meet, call, and chat to each other more frequently. In general, patients were matched to the same coach until the end of the intervention. However, if for some reason contact between dyads was interrupted prematurely (i.e., before 12-month follow-up), patients had the option to be re-matched. Dyads had an amount of €9,- to their disposal in order to support activities during each meeting.

The Forensic Network Coach is a trained volunteer who could be a role model stimulating participants to strengthen their social network, as well as a supportive social network member. The relationship between the patient-coach dyads is informal, meaning that coaches are non-professional volunteers not receiving payments for their services. Coaches received a training program that included (1) a three-hour practical training about a structured, goal-oriented social network protocol entitled ‘Natuurlijk, een netwerkcoach!’ [Of course, a network coach!] (57), (2) a nine-hour training focused on basic coaching skills for volunteer coaches, and (3) a two-hour informational training on forensic mental healthcare, providing coaching for forensic outpatients, and to reflect on the expectations, attitudes, and commitment of coaches. Noteworthy, coaches were instructed to focus first on developing a working alliance with the patient. Subsequently, they could use the abovementioned protocol as a tool to strengthen social networks. Coaches were encouraged to attend group supervision meetings or request individual supervision from the coordinator. Evaluation of FNC in the presence of the coordinator took place every 3–6 months and upon completion of FNC, after 12 months. Afterwards, patient-coach dyads could decide to stay connected without interference from De Regenboog Groep.

### Outcome measures

In this study, we examined the primary outcome measure and (key) secondary outcome measures, as detailed in the study protocol (50). An overview of the outcome variables, instruments, and assessment timepoints from baseline to the final follow-up assessment (i.e., at 18 months) can be found in the Supplementary Table S1.

The primary outcome was the mean difference in self-reported *mental wellbeing* between groups at 12-month follow-up (i.e., post-test assessment), which was measured with the Dutch version of the Mental Health Continuum - Short Form (MHC-SF) (58) from baseline to 18-month follow-up. Key secondary outcomes were the difference between groups from baseline to 18-month follow-up or the number of events within 12- and 18-month follow-up on (1) *psychiatric functioning* including (a) observer-rated general psychiatric functioning assessed with the Health of the Nations Outcome Scales (HoNOS) (59, 60), and (b) the number and duration of hospitalizations in addiction and/or psychiatric healthcare institutes based on self-report questions and data from the institutes’ official medical record, and (2) *criminal recidivism* defined as (a) the total number of rule-breaking and criminal behaviors in the past 6 months measured with a self-report delinquency scale (61) and (b) the number of incarcerations measured with self-report questions. Other secondary outcomes are (3) *social network* including (a) the self-reported size of the core social network and quality of social relationships in the core network measured with a self-developed interview based on the Modified Multiple Generator and Name Generator/Interpreter method (62, 63), (b) self-reported positive social support measured with the Social Support List - Interactions (64), and (c) self-reported loneliness measured with the Loneliness Scale (65), (4) self-reported number of days and quantity of *substance use* assessed with the Measurement in the Addictions for Triage and Evaluation 2.1 (66), (5) self-reported *quality of life* measured with the Dutch version of the Manchester Short Assessment of Quality of Life (55), and (6) observer-rated *self-sufficiency* assessed with the Dutch version of the Self-Sufficiency Matrix (54).

Additionally, demographic and patient characteristics (i.e., treatment histories, duration of outpatient care, and clinical diagnoses) were assessed with a self-developed questionnaire and obtained from the official medical record. The number of contact moments between participants and coaches of the FNC intervention group was documented. The duration of their contact (in months) was determined based on the official record of De Regenboog Groep. The duration of contact between dyads was used to define the per-protocol samples (i.e., no compliance, low compliance, and high compliance).

### Statistical methods

The statistical analyses were conducted in accordance with a prespecified analysis plan using IBM SPSS Statistics, version 27.0 (67).

Descriptive statistics were used to explore baseline characteristics. Differences in baseline characteristics between the two intervention groups were examined using chi-square tests for categorical variables and independent samples *t*-tests or Mann–Whitney U tests for continuous variables. Given the substantial dropout of participants in the intervention, between-group differences in duration of TAU from baseline to 12-, and 18-month follow-up were examined using Mann–Whitney U tests. Missingness of data points between participants in TAU+FNC and TAU was examined using a chi-square test. Furthermore, baseline characteristics of participants with and without two or more missing data points were compared, using chi-square tests for categorical variables and independent samples *t*-tests or Mann–Whitney U tests for continuous variables, to explore the differences between these participant groups.

Intention-to-treat analyses of the primary outcome measures and (key) secondary outcome measures were conducted, which included analyses of all the available data (between baseline and 18-month follow-up) from participants who completed baseline assessment (*N* = 102). In addition, per-protocol analyses were conducted on the primary (i.e., mental wellbeing) and key secondary outcomes only (i.e., general psychiatric functioning, hospitalization, criminal behavior, and incarceration), comparing different compliance groups of the FNC intervention (i.e., no compliance, low compliance, and high compliance) to the TAU intervention. Linear Mixed Models (LMM) analyses were used to analyze repeatedly measured continuous outcomes and normally distributed count outcomes. Generalized Estimating Equations (GEE) analyses were used to analyze repeatedly measured count outcomes with Poisson and negative binominal distributions, as well as one dichotomous outcome (i.e., quality of relationships with core social network members). For all LMM and GEE analyses, first the overall between-group treatment effect on average over time was analyzed and second, the between-group treatment effect at the different follow-up timepoints. For the latter, besides the intervention group variable (i.e., TAU+FNC versus TAU), also time (3-, 6-, 9-, 12-, and 18-month follow-up), and the group-by-time interactions were added to the model. Furthermore, Generalized Linear Models (GLM) was used to examine overall between-group treatment effect of the number and duration of hospitalization based on medical records obtained over the period from baseline to 12-month follow-up and over the 18-month follow-up period. All analyses were adjusted for the baseline value of the particular outcome and potential confounders (i.e., sex, age, ethnicity, type of forensic outpatient care site, and duration of outpatient treatment). In addition, for the outcomes of hospitalization, we adjusted for hospitalization at baseline.

Two-tailed significance level of *p* < 0.05 was used to assess the magnitude of treatment effects, Cohen’s *d* effect sizes were calculated for continuous outcomes by dividing the regression coefficients of an outcome by the standard deviation (SD) of the total outcome. Additionally, rate ratios (RR) were calculated for count outcomes and an odds ratio (OR) was calculated for the dichotomous outcome.

Finally, moderator analyses were conducted to explore moderators of treatment effects on primary and key secondary outcomes by adding a set of predefined potential moderators (i.e., males/females, age, comorbidity, comorbid intellectual disability, comorbid personality disorder, primary personality disorder, primary psychotic disorder, and primary substance use disorder) as interactions to the analyses.

## Results

### Baseline characteristics of participants

The participants included in the intention-to-treat sample (*N* = 102) were predominantly males (88.2%) between 17 and 67 years with a mean age of 40.5 years (*SD* = 12.8). The largest ethnic groups were white participants (40.2%) and black Caribbean or African participants (23.5%) respectively. Most participants were diagnosed with multiple disorders by clinicians (90.2%). Participants received mandatory treatment (59.8%) at baseline and reported histories of hospitalizations in addiction and/or psychiatric care institutes (63.7%). More baseline characteristics of participants are presented in Table 1. Demographic and clinical characteristics were well balanced between the groups at baseline assessment, with the exception of sex. There were slightly more females randomized to the TAU+FNC group (*n* = 10), compared to the TAU group (*n* = 3).

**Table 1:** Baseline characteristics.

	TAU + FNC (*n* = 51)	TAU (*n* = 51)	Total (*N* = 102)	Statistics
**Age, mean (SD)**	40.7 (12.7)	40.4 (13.0)	40.5 (12.8)	*t*(100) = 0.139, *p* = 0.890
**Sex, *n* (%)**				*X^2^*(1) = 6.044, *p* = 0.014*
Male	41 (45.6)	49 (96.1)	90 (88.2)	
Female	10 (19.6)	2 (3.9)	12 (11.8)	
**Ethnic origin, *n* (%)**				*X^2^*(3) = 2.001, *p* = 0.572
White	22 (43.1)	19 (37.3)	41 (40.2)	
Black Caribbean or African	9 (17.6)	15 (29.4)	24 (23.5)	
Arabic or North-African	9 (17.6)	8 (15.7)	17 (16.7)	
Asian^a^	1 (2.0)	1 (2.0)	2 (2.0)	
Multiple ethnic groups	10 (19.6)	8 (15.7)	18 (17.6)	
**Daily occupation, *n* (%)**				
Education	6 (11.8)	6 (11.8)	12 (11.8)	*X^2^*(1) = 0.000, *p* = 1.000
Paid employment	14 (27.5)	11 (21.6)	25 (24.5)	*X^2^*(1) = 0.477, *p* = 0.490
Supported activities	17 (33.3)	13 (25.5)	30 (29.4)	*X^2^*(1) = 0.756, *p* = 0.385
Other	16 (31.4)	23 (45.1)	39 (38.2)	*X^2^*(1) = 2.034, *p* = 0.154
**Income: receiving benefits, *n* (%)**	39 (76.5)	41 (80.4)	80 (78.4)	*X^2^*(1) = 0.232, *p* = 0.630
**Living situation, *n* (%)**				*X^2^*(2) = 2.326, *p* = 0.312
Alone	11 (21.6)	18 (35.3)	29 (28.4)	
With others^b^	13 (25.5)	9 (17.6)	22 (21.6)	
Supported housing^c^	23 (45.1)	23 (45.1)	46 (45.1)	
Other^a^	4 (7.8)	1 (2.0)	5 (9.8)	
**Highest education, *n* (%)**				*X^2^*(2) = 2.794, *p* = 0.247
Primary education (or no qualification)	16 (31.4)	12 (23.5)	28 (27.5)	
Preparatory secondary vocational education	20 (39.2)	16 (31.4)	36 (35.3)	
Senior secondary (vocational) education	10 (19.6)	20 (39.2)	30 (29.4)	
Higher professional/academic education^d^	4 (7.8)	2 (3.9)	6 (5.9)	
Unspecified^a^	1 (2.0)	1 (2.0)	2 (2.0)	
**Participants with partner, *n* (%)**	13 (25.5)	6 (11.8)	19 (18.6)	*X^2^*(1) = 3.169, *p* = 0.075
**Duration forensic outpatient care^e^, median (IQR)**	337.0 (231.0–1016.0)	352.0 (188.0–621.0)	344.5 (204.3–683.3)	*U* = 1446.5, *p* = 0.328
**Mandatory treatment, *n* (%)**	31 (60.8)	30 (58.8)	61 (59.8)	*X^2^*(1) = 0.041, *p* = 0.840
**Prior hospitalizations, *n* (%)**	31 (60.8)	34 (66.7)	65 (63.7)	*X^2^*(2) = 1.798, *p* = 0.407
Clinical addiction care	15 (48.4)	11 (32.4)	26 (40.0)	
Clinical psychiatric care	7 (22.6)	11 (32.4)	18 (27.7)	
Combination of clinical care	9 (29.0)	12 (35.3)	21 (32.3)	
**Primary clinical diagnosis, *n* (%)**				*X^2^*(2) = 0.879, *p* = 0.644
Substance use disorders	20 (39.2)	21 (41.2)	41 (40.2)	
Schizophrenia and psychotic spectrum disorders	8 (15.7)	11 (21.6)	19 (18.6)		
Bipolar and depressive disorders^f^	3 (5.9)	4 (7.8)	7 (6.9)		
Autism spectrum disorders^f^	3 (5.9)	4 (7.8)	7 (6.9)		
Personality disorders^f^	4 (7.8)	4 (7.8)	8 (7.8)		
Other	13 (25.5)	7 (13.7)	20 (19.6)		
**Comorbidity, *n* (%)**	45 (88.2)	47 (92.2)	92 (90.2)	*X^2^*(1) = 0.443, *p* = 0.505
Comorbid intellectual disability, *n* (%)	11 (21.6)	9 (17.6)	20 (19.6)	*X^2^*(1) = 0.249, *p* = 0.618
Comorbid personality disorder, *n* (%)	10 (19.6)	4 (7.8)	14 (13.7)	*X^2^*(1) = 2.981, *p* = 0.084
**Prior convictions, *n* (%)**	43 (84.3)	42 (82.4)	85 (83.3)	*X^2^*(3) = 0.354, *p* = 0.950
No prior convictions	8 (15.7)	8 (15.7)	17 (16.7)	
One or two	10 (19.6)	9 (17.6)	19 (18.6)	
Three to ten	16 (31.4)	18 (35.3)	34 (33.3)	
More than ten	17 (33.3)	15 (29.4)	32 (31.4)	

TAU, treatment as usual; FNC, forensic network coaching; SD, standard deviation; IRQ, interquartile range. ^a^Category not included in the analyses. ^b^Others include parents, partner and/or children. ^c^Category include mental healthcare institutes. ^d^Category was added to ‘Senior secondary (vocational) education’ in the analyses. ^e^Duration of outpatient care at Inforsa in days. ^f^Category was added to ‘Other’ in the analyses. *p < 0.05.

In addition, the duration of TAU was not significantly different between treatment groups at 12-months follow-up (Mann–Whitney *U* = 980.5, *p* = 0.757), as well as at 18-months follow-up (Mann–Whitney *U* = 894.0, *p* = 0.802). Participants in the TAU+FNC group received TAU for an average of 9.9 months (SD = 3.5) at 12-month follow-up and 12.8 months (SD = 5.9) at 18-month follow-up. Participants in the TAU group received TAU for an average of 10.2 months (SD = 3.1) at 12-month follow-up and 13.4 months (SD = 5.5) at 18-month follow-up. On average, patient-coach dyads were matched 7.3 months (SD = 3.6) from baseline to 12-month follow-up and 9.0 months (SD = 4.8) from baseline to 18-month follow-up. Furthermore, the percentage of participants with missingness on two or more follow-up assessments (i.e., missingness at 3-, 6-, 9-, 12-, and/or 18-months) was not significantly different between treatment groups. In the TAU+FNC group 78% of the participants (*n* = 40) completed two or more follow-up assessments, compared to 74% in the TAU group (*n* = 38). In addition, comparison of demographic and clinical baseline characteristics of participants with two or more missing data points and participants with less than two missing data points showed that missingness was associated with sex; 53 % of female participants (*n* = 7) and 18.9% of the males (*n* = 17) missed two or more data points (*p* = 0.006) and education; 36.1% of participants with senior secondary and higher education (*n* = 13), 19.4% of participants with preparatory secondary education (*n* = 7), and 10.7% of participants with primary education or no qualifications (*n* = 3) missed two or more data points (*p* = 0.046).

### Treatment effects of intention-to-treat analyses

A complete overview of the adjusted treatment effects from the intention-to-treat analyses of our primary and (key) secondary outcomes and the effects at 12- and 18-month follow-up are presented in Table 2. Results of crude and adjusted treatment effects on primary and (key) secondary outcomes, including all follow-up assessments (i.e., 3-, 6-, and 9-month follow-up), can be found in the Supplementary Table S2. Regarding mental wellbeing, in general, non-significant negative regression coefficients were found, indicating that treatment effects among participants in TAU+FNC compared to TAU did not differ significantly on average over time (adjusted mean difference = −0.193, 95% CI -0.434 to 0.047, *p* = 0.114). Furthermore, inspection of between-group treatment effects at different timepoints revealed significant lower mental wellbeing in TAU+FNC participants compared to TAU at 6-month follow-up (adjusted mean difference = −0.337, 95% CI -0.674 to 0.000, *p* = 0.050). Yet none of the effects were significant at other timepoints, including our primary effect of interest at 12-month follow-up (adjusted mean difference = −0.151, 95% CI -0.484 to 0.182, *p* = 0.373).

**Table 2:** Treatment effects on primary and secondary outcomes from intention-to-treat analyses (*N* = 102).

		Descriptive statistics of raw data		Adjusted effects	
Outcome variables (measurement type)	Assessment time	TAU + FNC group	TAU group	Between-group effect	Standardized effect size
**Primary outcome**					
Mental wellbeing (self-report)^a^				−0.193 (−0.434 to 0.047)^d^	−0.187
	Baseline	3.797 (0.967), *n* = 50	3.622 (1.146), *n* = 51		
	12 months	3.993 (0.926), *n* = 42	4.033 (1.196), *n* = 42	−0.151 (−0.484 to 0.182)	−0.142
	18 months	3.953 (0.918), *n* = 39	3.948 (1.062), *n* = 39	−0.234 (−0.578 to 0.111)	−0.236
**Key secondary outcomes**					
Psychiatric functioning					
General psychiatric functioning (observer-rated)^a^				−0,009 (−0.139 to 0.120)^d^	−0.020
	Baseline	0.866 (0.434), *n* = 51	0.891 (0.445), *n* = 51		
	12 months	0.772 (0.450), *n* = 42	0.764 (0.506), *n* = 42	−0.070 (−0.243 to 0.103)	−0.147
	18 months	0.757 (0.457), *n* = 37	0.785 (0.487), *n* = 38	−0.067 (−0.246 to 0.113)	−0.143
Hospitalization					
Number in addiction and psychiatric care (self-report)^b^				0.976 (0.488 to 1.952)^d^	NA
	12 months	0.07 (0.261), *n* = 42	0.12 (0.400), *n* = 41	0.593 (0.133 to 2.647)	NA
	18 months	0.22 (0.584), *n* = 37	0.15 (0.362), *n* = 40	1.635 (0.558 to 4.790)	NA
Number in internal mental healthcare institute (medical record)^b^					
	12 months	0.167 (0.476), *n* = 48	0.192 (0.495), *n* = 47	1.020 (0.304 to 3.424)	NA
	18 months	0.271 (0.644), *n* = 48	0.234 (0.520), *n* = 47	1.016 (0.357 to 2.890)	NA
Days in internal mental healthcare institute (medical record)^b^					
	12 months	3.354 (9.486), *n* = 48	24.319 (78.536), *n* = 47	0.483 (0.252 to 0.926)*	NA
	18 months	5.000 (13.358), *n* = 48	33.851 (107.420), *n* = 47	0.244 (0.130 to 0.455)***	NA
Criminal recidivism					
Criminal behavior (self-report)^b^				0.346 (0.152 to 0.787)^d^*	NA
	Baseline	47.765 (103.125), *n* = 51	13.286 (46.142), *n* = 49		
	12 months	26.738 (55.231), *n* = 42	28.275 (79.831), *n* = 40	0.575 (0.225 to 1.470)	NA
	18 months	19.054 (50.862), *n* = 37	28.622 (59.668), *n* = 37	0.180 (0.053 to 0.611)**	NA
Incarceration (self-report)^b^				0.451 (0.172 to 1.183)^d^	NA
	12 months	0.07 (0.261), *n* = 42	0.12 (0.510), *n* = 41	0.670 (0.136 to 3.292)	NA
	18 months	0.00 (0.000), *n* = 37	0.15 (0.366), *n* = 39	NA	NA
**Other secondary outcomes**					
Social network (self-report)					
Core social network					
Size^a^				0.377 (−0.299 to 1.053)^d^	0.202
	Baseline	3.941 (2.204), *n* = 51	4.078 (3.149), *n* = 51		
	12 months	5.210 (2.928), *n* = 41	3.825 (2.561), *n* = 40	0.741 (−0.052 to 1.535)	0.368
	18 months	4.324 (2.583), *n* = 37	4.158 (2.400), *n* = 38	−0.018 (−0.831 to 0.794)	−0.011
Quality^c^				1.061 (0.476 to 2.362)^d^	NA
	Baseline	21 (41.2%), *n* = 51	23 (46.0%), *n* = 50		
	12 months	19 (46,3%), *n* = 41	21 (52.5%), *n* = 40	1.326 (0.497 to 3.539)	NA
	18 months	20 (54.1%), *n* = 37	19 (50.0%), *n* = 38	0.834 (0.310 to 2.240)	NA
Positive social support^a^				0.130 (−0.042 to 0.302)^d^	0.238
	Baseline	2.163 (0.521), *n* = 51	2.090 (0.563), *n* = 51		
	12 months	2.235 (0.530), *n* = 42	2.080 (0.557), *n* = 41	0.138 (−0.054 to 0.330)	0.253
	18 months	2.243 (0.512), *n* = 37	2.110 (0.584), *n* = 39	0.121 (−0.076 to 0.318)	0.220
Loneliness^a^				−0.078 (−0.349 to 0.192)^d^	−0.090
	Baseline	3.218 (0.803), *n* = 51	3.410 (0.769), *n* = 51		
	12 months	2.794 (0.774), *n* = 42	3.031 (0.978), *n* = 41	−0.162 (−0.478 to 0.153)	−0.183
	18 months	2.786 (0.831), *n* = 37	2.907 (0.862), *n* = 39	0.013 (−0.311 to 0.338)	0.015
Substance use (self-report)					
Quantity alcohol^b^				1.078 (0.682 to 1.702)^d^	NA
	Baseline	9.588 (14.054), *n* = 51	6.902 (12.911), *n* = 51		
	12 months	7.143 (9.651), *n* = 42	5.857 (9.527), *n* = 42	0.750 (0.371 to 1.517)	NA
	18 months	5.684 (10.419), *n* = 38	4.135 (8.011), *n* = 37	0.876 (0.398 to 1.930)	NA
Quantity cannabis^b^				1.175 (0.726 to 1.902)^d^	NA
	Baseline	0.510 (0.987), *n* = 51	0.510 (0.834), *n* = 51		
	12 months	0.405 (0.828), *n* = 42	0.524 (0.917), *n* = 42	0.697 (0.306 to 1.585)	NA
	18 months	0.526 (0.862), *n* = 38	0.432 (0.765), *n* = 37	1.136 (0.566 to 2.279)	NA
Quantity hard drugs^b^				0.885 (0.320 to 2.451)^d^	NA
	Baseline	0.216 (0.757), *n* = 51	0.118 (0.475), *n* = 51		
	12 months	0.238 (0.532), *n* = 42	0.643 (3.091), *n* = 42	0.356 (0.103 to 1.232)	NA
	18 months	0.105 (0.311), *n* = 38	0.162 (0.602), *n* = 37	0.725 (0.157 to 3.357)	NA
Days alcohol^b^				0.982 (0.600 to 1.608)^d^	NA
	Baseline	6.647 (9.492), *n* = 51	6.137 (9.633), *n* = 51		
	12 months	6.191 (9.253), *n* = 42	4.310 (7.588), *n* = 42	1.213 (0.586 to 2.514)	NA
	18 months	6.368 (9.604), *n* = 38	4.028 (7.606), *n* = 36	0.951 (0.452 to 2.001)	NA
Days cannabis^b^				1.199 (0.680 to 2.112)^d^	NA
	Baseline	9.000 (12.291), *n* = 51	8.765 (11.911), *n* = 51		
	12 months	8.857 (12.215), *n* = 42	5.000 (9.571), *n* = 42	1.650 (0.706 to 3.859)	NA
	18 months	10.342 (13.358), *n* = 38	6.108 (11.007), *n* = 37	0.741 (0.318 to 1.724)	NA
Days hard drugs^b^				0.693 (0.278 to 1.727)^d^	NA
	Baseline	2.078 (5.932), *n* = 51	0.412 (1.780), *n* = 51		
	12 months	0.810 (3.133), *n* = 42	1.071 (3.502), *n* = 42	0.418 (0.077 to 2.274)	NA
	18 months	2.237 (6.158), *n* = 38	0.405 (1.423), *n* = 37	1.123 (0.285 to 4.419)	NA
Quality of life (self-report)^a^				−0.147 (−0.499 to 0.204)^d^	−0.126
	Baseline	4.172 (1.106), *n* = 51	4.029 (0.964), *n* = 51		
	12 months	4.611 (1.195), *n* = 42	4.701 (1.110), *n* = 42	−0.112 (−0.556 to 0.333)	−0.098
	18 months	4.432 (1.319), *n* = 39	4.540 (1.066), *n* = 39	−0.322 (−0.776 to 0.132)	−0.270
Self-sufficiency (observer-rated)^a^				0.023 (−0.124 to 0.169)^d^	0.046
	Baseline	3.440 (0.395), *n* = 51	3.424 (0.346), *n* = 51		
	12 months	3.575 (0.542), *n* = 42	3.581 (0.504), *n* = 42	0.070 (−0.114 to 0.254)	0.135
	18 months	3.611 (0.556), *n* = 37	3.623 (0.492), *n* = 38	0.019 (−0.171 to 0.209)	0.036

Data were analyzed with LMM/GEE/GLM and presented as adjusted mean differences (95% CI) with standardized effect sizes for continuous outcomes^a^, rate ratios (95% CI) for count outcomes^b^, and odds ratio (95% CI) for the dichotomous outcome^c^;

^d^Between-group effect on average over time.

*p < 0.05;

**p < 0.01;

***p < 0.001.

From the results of the key secondary outcomes related to psychiatric functioning in Table 2, it is apparent that significant between-group treatment effects were found on duration of hospitalization in the internal mental healthcare institute within 12-month follow-up, as well as 18-months follow-up. Participants in the TAU group were hospitalized more days, compared to participants in the TAU+FNC group. Specifically, participants in the TAU group were hospitalized 2.1 times more days within 12-months (RR_TAU+_ = 0.483, 95% CI 0.252 to 0.926, *p* = 0.028), and 4.1 times more days within 18-months (RR_TAU+_ = 0.244, 95% CI 0.130 to 0.455, *p* < 0.001), compared to TAU+FNC participants. Furthermore, no significant treatment effects were found on the number of hospitalizations in the internal mental healthcare institute, the number of hospitalizations in addiction and psychiatric care, and general psychiatric functioning.

For criminal recidivism, significant between-group treatment effects favoring the TAU+FNC group were found on criminal behavior. The effect on the number of criminal behaviors on average over time indicated that participants in the TAU group reported 2.9 times more criminal behaviors compared to TAU+FNC participants (RR_TAU+_ = 0.346, 95% CI 0.152 to 0.787, *p* = 0.011). At 18-month follow-up, TAU participants showed 5.6 times more criminal behaviors compared to TAU+FNC participants (RR_TAU+_ = 0.180, 95% CI 0.053 to 0.611, *p* = 0.006). The treatment effect on criminal behavior at 12-month follow-up indicated no significant difference between TAU and TAU+FNC participants. Furthermore, no significant treatment effects were found on incarceration. Noteworthy, the between-group effect of the number of incarcerations at 18-month follow-up could not be analyzed due to an overrepresentation of 0-values in the outcome variable.

Regarding the other secondary outcomes, no significant between-group treatment effects were found on social network outcomes, such as network size, the availability of high quality relationships in the core social network, positive social support, and loneliness, both on average over time and at different timepoints. Furthermore, also results of substance use generally showed no significant between-group treatment effects on average over time and at different timepoints. Noteworthy, inspection of effects on hard drug use at different timepoints (Supplementary Table S2) revealed a significant treatment effect on hard drug use at 6-month follow-up in favor of TAU participants. The results indicate that participants in the TAU+FNC group reported 6.2 times more hard drug use on average at 6-month follow-up, compared to TAU (RR_TAU+_ = 6.159, 95% CI 1.177 to 32.238, *p* = 0.031). Lastly, no significant between-group treatment effects were found on quality of life and self-sufficiency (Table 2).

### Treatment effects of per-protocol analyses

A complete overview of the adjusted treatment effects on the primary and key secondary outcomes, comparing the different compliance groups to TAU in the per-protocol analyses, are presented in Table 3. In general, the per-protocol analyses produced similar results as the intention-to-treat analyses, revealing no differential treatment effects on the primary outcome mental wellbeing and positive treatment effects on key secondary outcomes (i.e., duration of hospitalization in the internal mental healthcare institute and criminal behavior) in participants of the high compliance group compared to TAU. Further inspection of treatment effects in the different compliance groups showed significantly higher positive treatment effects on duration of hospitalization in the internal mental healthcare institute in high compliance participants compared to TAU. However, for criminal behavior, positive treatment effects in no compliance participants were slightly higher than in high compliance participants, although both were significantly better than TAU. Overall, no clear evidence of dose–response effects were found, as treatment effects of duration of hospitalization and criminal behavior did not increase as compliance increased.

**Table 3:** Treatment effects on primary and key secondary outcomes from per-protocol analyses comparing compliance groups to TAU from baseline to 18-month follow-up (*N* = 102).

	TAU + FNC groups		
Outcome variables (measurement type)	No compliance	Low compliance	High compliance
**Primary outcome**			
Mental wellbeing (self-report)^a^	−0.201 (−0.545 to 0.142)	−0.341 (−0.685 to 0.003)	−0.070 (−0.392 to 0.253)
**Key secondary outcomes**			
Psychiatric functioning			
General psychiatric functioning (observer-rated)^a^	−0.092 (−0.269 to 0.085)	0.137 (−0.048 to 0.321)	−0.054 (−0.227 to 0.119)
Hospitalization			
Number in addiction and psychiatric care (self-report)^b^	1.277 (0.573 to 2.848)	1.184 (0.513 to 2.728)	0.425 (0.090 to 2.001)
Number in internal mental healthcare institute at 12 months (medical record)^b^	1.381 (0.340 to 5.604)	0.452 (0.044 to 4.660)	0.938 (0.154 to 5.703)
Number in internal mental healthcare institute at 18 months (medical record)^b^	1.254 (0.358 to 4.392)	1.428 (0.319 to 6.385)	0.513 (0.093 to 2.837)
Days in internal mental healthcare institute at 12 months (medical record)^b^	0.481 (0.239 to 0.970)*	1.212 (0.351 to 4.183)	0.293 (0.099 to 0.866)*
Days in internal mental healthcare institute at 18 months (medical record)^b^	0.261 (0.130 to 0.524)***	1.192 (0.331 to 4.293)	0.081 (0.029 to 0.229)***
Criminal recidivism			
Criminal behavior (self-report)^b^	0.206 (0.067 to 0.628)**	0.629 (0.227 to 1.740)	0.237 (0.060 to 0.933)*
Incarceration (self-report)^b^	0.923 (0.319 to 2.669)	0.262 (0.063 to 1.091)	0.133 (0.017 to 1.033)

Data were analyzed with LMM/GEE/GLM and presented as adjusted mean differences (95% CI) for continuous outcomes^a^ and rate ratios (95% CI) for count outcomes^b^. TAU = reference category.

* *p* < 0.05;

** *p* < 0.01;

*** *p* < 0.001.

### Moderators of treatment effects

The results of our moderator analyses should be considered exploratory due to the lack of statistical power. Exploration of potential moderators showed that most of the variables (i.e., sex, age, primary personality disorder, and primary psychotic disorder) did not modify treatment effects. We did find significant effect modification of the variables primary substance use disorder, comorbidity, and sex. Stronger treatment effects were found for TAU+FNC participants without primary substance use disorders on the duration of hospitalization within 12-month follow-up (RR_TAU+_ = 0.016, 95% CI 0.006 to 0.014), as well as within 18-month follow-up (RR_TAU+_ = 0.013, 95% CI 0.005 to 0.033), compared to TAU participants. In contrast, negative effects were found for the group with primary substance use disorders (12-month RR_TAU+_ = 1.513, 95% CI 0.583 to 3.929; 18-month RR_TAU+_ = 2.019, 95% CI 0.780 to 5.227). Furthermore, the effects on duration of hospitalization within 18-month follow-up were stronger for TAU+FNC participants with comorbid disorders compared to TAU (RR_TAU+_ = 0.078, 95% CI 0.037 to 0.166). Again, negative effects were found in those without comorbid disorders (RR_TAU+_ = 1.426, 95% CI 0.320 to 6.346). Lastly, stronger treatment effects on criminal behavior were found for male participants receiving TAU+FNC compared to TAU (RR_TAU+_ = 0.519, 95% CI 0.203 to 1.330). The effects were negative in female participants (RR_TAU+_ = 13.885, 95% CI 2.090 to 92.253). Noteworthy, the effects of various potential moderating variables on the number and duration of hospitalization and incarceration could not be estimated due to an excess of 0-values of the outcome in categories of potential moderators.

## Discussion

There is an urgent and ongoing need to develop and improve effectiveness of evidence-based interventions for forensic psychiatric patients (10–13). A supportive social network is considered an important protective factor that may improve mental health outcomes and reduce criminal recidivism in forensic psychiatric care (14–16). Yet, the effectiveness of interventions aimed at promoting supportive social networks among forensic psychiatric patients has remained unclear. Therefore, in this RCT we examined whether an additive informal social network intervention, provided by trained volunteer coaches in the community, could improve treatment outcomes among outpatients receiving forensic psychiatric care. To this end, we aimed to (1) examine treatment effects of patients receiving an additive social network intervention versus treatment as usual alone on a broad range of outcomes, including mental wellbeing (primary outcome), general psychiatric functioning, number and duration of hospitalization, criminal behavior, incarceration (key secondary outcomes), and other relevant treatment outcomes, on average over time and at different timepoints from baseline assessment to 18-months follow-up, (2) explore treatment effects of patients with either no, low, or high compliance to the additive intervention on mental wellbeing, psychiatric functioning, and criminal recidivism on average over time to determine dose–response effects, and (3) explore potential moderators of treatment effects to gain insight into which patients might benefit more from the additive intervention.

With respect to the first aim, contrary to expectations, the current study found no significant differential effects of the additive intervention on mental wellbeing on average over time and at different timepoints, with the exemption of the 6-month follow-up. Surprisingly, at 6-months, mental wellbeing in patients receiving the additive intervention was lower compared to patients receiving TAU. However, in line with our expectations, significant benefits were found on duration of hospitalization and criminal behavior. Specifically, the findings show that patients receiving TAU were hospitalized 2.1 times more days within 12 months after baseline, compared to patients in the additive intervention, and that this effect increased over 18 months. Patients receiving TAU reported 2.9 times more criminal behaviors on average over time, with a stronger effect when groups were compared at 18 months. Furthermore, no benefits of the additive intervention on other treatment outcomes (i.e., social network, substance use, quality of life, and self-sufficiency) were found. On the contrary, besides mental wellbeing, hard drugs use at 6-month follow-up was temporary deteriorated in patients receiving the additive intervention, compared to patients receiving TAU. Next, exploration of the effects across patients with different levels of compliance to the additive intervention showed no evidence of dose–response effects. Only for duration of hospitalization, stronger differential benefits were found in patients who fully adhered to the intervention. However, caution must be applied, as the groups of patients with different compliance levels were small. Lastly, the exploratory findings from moderator analyses suggested that the treatment effects of the additive intervention may only be positive for patients with no primary substance use disorders, patients with comorbid disorders, and males.

There are several possible explanations for our findings. The null findings on mental wellbeing could indicate more limited benefits of informal social network interventions in psychiatric patients with severe problems and complex needs, consistent with a recent study among patients with schizophrenia as well as with our meta-analysis of general social network interventions for psychiatric populations, both of which found no improvements on mental wellbeing (41, Swinkels, Hoeve et al. submitted). Furthermore, a befriending study including patients with SMI, a population comparable to the forensic population, demonstrated that mental wellbeing only increased in patients with significant improvements on social support (68). As we found no significant improvements on social network and support outcomes in our study, the null findings on mental wellbeing might be explained by the absence of clear improvements on social network and support outcomes. Another explanation for the findings, including the temporary negative effects on mental wellbeing at 6 months, may be related to the overall low compliance of patients in our trial. Regarding the negative effects, for example, it is possible that patients with low compliance had more severe mental health problems and complex needs (69). Furthermore, although speculative, the failure to participate in the intervention and to develop a bond with the volunteer could cause frustrations, disappointment, or self-doubt. Yet, previous studies among general psychiatric populations also faced compliance difficulties but did not find negative effects, which could suggest that effects of informal social network interventions might be different in forensic psychiatric populations (41, 68). Probably, even higher compliance rates and longer timeframes are needed to demonstrate improvements on mental wellbeing among forensic patients (35, 68). On the other hand, we cannot rule out the influence of contextual factors, such as the COVID-19 pandemic, relationship problems, financial problems, and unstable housing. These factors may have complicated the contact between patient-coach dyads and prevented them from participating in social activities in the community, impeding the effects (69, 70).

Additionally, we found positive effects on duration of hospitalization and criminal behavior in patients receiving the informal social network intervention in addition to TAU. Moreover, these findings appeared to be robust, as intention-to-treat and per-protocol analyses generally yielded similar results. This study is the first to demonstrate positive effects of an additive informal social network interventions on hospitalization. Similar results have been demonstrated in other additive peer mentoring interventions that shared the primary aim of providing support to patients with SMI (71–73). Based on these studies, we hypothesize that the provision of additional social support by volunteers during treatment might enhance treatment adherence and a sense of self-efficacy of patients, reducing hospitalization and criminal recidivism (71, 73, 74). Furthermore, the positive findings on criminal behavior found in the current study are consistent with previous meta-analyses and RCTs on mentoring interventions among forensic populations (43–46).

Interestingly, the per-protocol analyses showed that effects on criminal behavior were similar, and even slightly stronger, in patients who did not start with the additive intervention, compared to those in patients fully adhering. Based on our qualitative study, in which patients who were unwilling to start reported that they had enough social relationships and that they did not need assistance to improve their social network, we hypothesize that patients with no compliance could have been more self-sufficient in developing and maintaining social connections, as they refused to start with the intervention (69). Future research on patient characteristics between compliance groups, which was beyond the scope of the current study, should aim to reveal which patients are more likely to adhere to and benefit from the intervention.

### Strengths and limitations

This study has several strengths. To the best of our knowledge, this is the first study to examine an additive informal social network intervention among forensic psychiatric outpatients with complex needs. A rigorous study design was used, as we were able to examine a relatively large forensic population in an RCT with multiple measurements over a long follow-up period (18 months). Furthermore, a broad range of treatment outcomes were examined including various sources, such as self-report and observer-rated instruments, as well as official medical records. The study was conducted in day-to-day clinical practice, which increased the ecological validity and generalizability of the study methods and results. Moreover, the overall study dropout rate was lower than expected (i.e., 21%) and the overall response rates on the six assessments from baseline to 18 months follow-up was evenly distributed between the treatment groups (i.e., 79% in TAU+FNC vs. 82% in TAU), minimizing bias due to missing outcome data. The informal social network intervention was developed based on clearly defined aspects of befriending and mentoring programs, and delivered by an informal care institute with longstanding experience in the delivery of volunteer-based interventions (i.e., recruitment, training, matching, and supervision of volunteers).

This study also has a number of limitations. Despite its rigorous design and the relatively large forensic population we were able to include, this study is still a relative small trial. Thus, findings of this study should be interpreted with caution and more research is recommended to confirm these results. As mentioned, the overall engagement of patients in the intervention was low (although expected) and varied over the 18-month follow-up period, which probably affected treatment efficacy. Furthermore, we included a group of patients with a wide range of psychiatric diagnoses, which complicates interpretation of the findings across diagnostic groups. There were challenges in the implementation of the intervention that may also hinder applicability, such as the availability of volunteers who matched the specific characteristics and needs of patients, preventing patients to start with the intervention conform protocol (i.e., within 2 months after their baseline assessment). Besides, the goal-oriented intervention protocol ‘Of course, a network coach!’ was not often used and was not considered feasible (69). Moreover, patients were found unwilling or unable to (timely) start with the intervention. Consequently, the overall treatment effects (i.e., intention-to-treat effects) should be interpreted with caution, as these effects include considerable different levels of compliance and cannot be extrapolated to the sample as a whole. Therefore, the effects across patients with different levels of compliance provided a more realistic insight about treatment effectiveness.

In addition, regarding the measurement of outcomes, caution must be applied to our measurement of criminal recidivism. In the current study, we used self-reported data to define criminal recidivism, which may be sensitive to under- and overreporting, raising questions about the validity of our findings (61, 75). It was not feasible to collect and define criminal recidivism based on official data from the Police Information Service and Justice Documentation System, due to time constraints and the long processing time required to obtain valid data, respectively. However, previous research demonstrated that all measures of criminal recidivism have limitations and should therefore preferably be used conjointly (61, 75). Efforts were made to minimize response bias by conducting one-to-one assessments, emphasizing patient privacy and confidentiality, and choosing a short response period of 6 months. Nevertheless, the overdispersion observed in the outcome of criminal behavior, although present in both treatment groups, could be an indication of overreporting. Furthermore, the outcomes of hospitalization from official medical records (i.e., number and days) could be limited, as these outcomes are based solely on hospitalizations within the mental healthcare institute where this study was conducted.

Finally, we want to acknowledge the ethical concerns and the corresponding deviations from the intended interventions that may arose because of the trial context. We compared TAU with an additive informal social network intervention to TAU alone in a complex patient group with limited social support, which could have caused disappointment in patients allocated to the TAU arm of the trial. However, participants received information about the randomization procedure beforehand and agreed with the conditions of the trial, including the possibility to be allocated to TAU. Although we cannot completely rule out this bias, we did not encounter higher study dropout rates in TAU. In contrast, slightly more dropout was found in the additive intervention group (i.e., 24% in TAU+FNC vs. 18% in TAU). Furthermore, on the ethical aspect, clinicians of patients in both treatment groups were regularly (i.e., after each follow-up assessments) urged by the researchers to discuss social network-related needs with their patients.

### Implications for research and practice

Regardless of the limitations, informal social network interventions are accessible and low-cost interventions that could provide a meaningful addition to current clinical practice for forensic outpatients with complex needs given its potential to improve treatment outcomes of hospitalization and criminal recidivism. Evidently, this is not a one-fits-all intervention, as the compliance as well as the effects may vary between individual patients (69). Therefore, a flexible implementation responding to patients’ abilities and needs, such as the frequency and type of contact, duration of the intervention period (i.e., open-ended or not), and the personalized process of matching patients to volunteers based on preferences and characteristics, seemed important requirements. In our trial setting, in which patients had to be matched within a preferred timeframe and patient-coach dyads were intended to meet regularly over a period of 12 months, it was more complicated to respond to patient preferences. Clinical settings allow for a more flexible implementation. Therefore, outcomes and compliance levels might improve in clinical settings. Furthermore, based on our exploratory findings from moderator analyses, we cannot yet determine which patients are more likely to benefit from the additive intervention. Nevertheless, our findings could encourage clinicians to explore its potential for individual patients, particularly in patients without primary substance use disorders, patients with comorbid disorders, and males.

Future research is needed to examine how effects of informal social network interventions can be further improved and which forensic psychiatric patients might benefit from additive informal social network interventions. Moreover, research is recommended to determine how to adapt these interventions for subgroups of patients with unfavorable outcomes found in our study (i.e., patients with substance use disorders, patients without comorbid disorders, and females). It would be worth exploring whether the effectiveness could be improved by extending the duration of the intervention, allowing patients and volunteers more time to develop a supportive relationship. However, we do acknowledge that it can be difficult to recruit volunteers willing to commit for an extended period. To accomplish reliable evidence, more large-scale (e.g., multi-center) clinical trials collecting detailed program integrity data, intervention characteristics, and patient characteristics should be conducted; a challenging task. As discussed by researchers of a previous relatively large RCT that examined the effectiveness of a volunteer-based social network intervention, it should be considered whether an RCT design is the most appropriate method (41). RCTs among (forensic) patients with SMI are time-consuming because these studies require long inclusion periods to obtain sufficiently large samples and a great deal of effort must be invested in data collection to avoid attrition at assessments over time. Moreover, previous studies have shown compliance problems similar to those in our study, which makes it even more difficult to achieve sufficient samples that lead to solid conclusions (38, 41, 68). More stringent study procedures, for example, inclusion and random allocation of patients who are socially isolated and/or highly motivated to be matched to a volunteer, might raise ethical concerns. Alternatively, randomized encouragement trials, in which participants are randomly assigned to a treatment and can choose whether or not they want to receive the allocated treatment, or matched case–control studies, could be considered to increase compliance (76). In addition, the use of multiple methods to examine effectiveness of these complex social network interventions in forensic populations, including quantitative as well as qualitative methods, is encouraged. For quantitative outcomes, we recommend combining self-report, observer-rated, and official data sources. Further research could also include information from different perspectives, such as clinicians, romantic partners, family, and friends. Lastly, given our positive effects on duration of hospitalization and criminal behavior in outpatients, another promising line of research would be to examine the effects of the additive intervention among forensic psychiatric inpatients who may go on leave or are reentering the community after clinical treatment. Volunteers could prepare patients for reentry in the community, as shown in previous studies among reentering prisoners (45–47).

## Conclusion

There is an urgent and ongoing need to develop and improve evidence-based interventions for forensic psychiatric patients with complex needs (10–13). This is the first study to examine whether an additive informal social network intervention could improve treatment outcomes among forensic outpatients. Although no benefits were found on the primary outcome of mental wellbeing, the findings suggest that the additive intervention improved other relevant treatment outcomes, such as the duration of hospitalization and criminal behavior. Noteworthy, no evidence of dose–response effects were found. We did find evidence of stronger effects in patients in specific subgroups, such as patients without primary substance use disorders, with comorbid disorders, and males. On the other hand, effects may be adverse in patients who fail to adhere, patients with primary substance use disorders, patients without comorbid disorders, and females. In sum, these findings suggest that forensic psychiatric treatment for outpatients can be optimized by collaborating with informal care initiatives aimed at improving supportive social networks within the community. Future research should further explore which patients might benefit from additive informal social network interventions and determine whether longer timeframes and better patient adherence will lead to sustainable supportive social networks and improved mental wellbeing.

## Data availability statement

The raw data supporting the conclusions of this article will be made available by the authors, without undue reservation.

## Ethics statement

The studies involving human participants were reviewed and approved by the Medical Ethics Committee VU Medical Center (METc VUmc). The patients/participants provided their written informed consent to participate in this study.

## Author contributions

LS, TP, JH, JD, and AP were involved in the design of the study. LS was responsible for the project management, including the implementation, recruitment, data collection, data analysis, and writing of the first draft of the manuscript, under the supervision of AP, JD, and TP. JT was involved in the data analysis and verified the final analyses. All authors contributed to the article and approved the submitted version.

## Funding

This study was funded by the Stichting tot Steun VCVGZ, a non-commercial organization, under Grant number 230. Personnel costs of researchers are partially funded by the trial sponsor, Inforsa Forensic Mental Healthcare. The funding agencies were not involved in the planning of the study design, data collection, data analysis, and decisions to publish or preparation of the manuscript.

## Conflict of interest

The authors declare that the research was conducted in the absence of any commercial or financial relationships that could be construed as a potential conflict of interest.

## Publisher’s note

All claims expressed in this article are solely those of the authors and do not necessarily represent those of their affiliated organizations, or those of the publisher, the editors and the reviewers. Any product that may be evaluated in this article, or claim that may be made by its manufacturer, is not guaranteed or endorsed by the publisher.
