# Supplementary material for: The effectiveness of an additive informal social network intervention for forensic psychiatric outpatients: results of a randomized controlled trial
Source: Front Psychiatry. 2023 May 24;14:1129492. doi: 10.3389/fpsyt.2023.1129492 (PMC10244564; doi:10.3389/fpsyt.2023.1129492)
Supplement: Supplementary file 1 [file Table_1.docx]

Supplementary Material

The effectiveness of an additive informal social network intervention for forensic psychiatric outpatients: results of a randomized controlled trial

**Lise T. A. Swinkels*, Thimo M. van der Pol, Jos Twisk, Janna F. ter Harmsel, Jack J. M. Dekker, Arne Popma**

***Correspondence:** Lise Swinkels: [lise.swinkels@inforsa.nl](mailto:lise.swinkels@inforsa.nl)

#
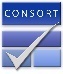
CONSORT 2010 checklist

**CONSORT 2010 checklist of information to include when reporting a randomised trial***

| **Section/Topic** | **Item No** | **Checklist item** | **Reported on page No** |
| --- | --- | --- | --- |
| **Title and abstract** | | | |
|  | 1a | Identification as a randomised trial in the title | 1 |
|  | 1b | Structured summary of trial design, methods, results, and conclusions (for specific guidance see CONSORT for abstracts) | 3 |
| **Introduction** | | | |
| Background and objectives | 2a | Scientific background and explanation of rationale | 2 |
|  | 2b | Specific objectives or hypotheses | 3 |
| **Methods** | | | |
| Trial design | 3a | Description of trial design (such as parallel, factorial) including allocation ratio | 3 |
|  | 3b | Important changes to methods after trial commencement (such as eligibility criteria), with reasons | NA |
| Participants | 4a | Eligibility criteria for participants | 3 |
|  | 4b | Settings and locations where the data were collected | 3 |
| Interventions | 5 | The interventions for each group with sufficient details to allow replication, including how and when they were actually administered | 5 |
| Outcomes | 6a | Completely defined pre-specified primary and secondary outcome measures, including how and when they were assessed | 6, Supplementary Material (Table 1) |
|  | 6b | Any changes to trial outcomes after the trial commenced, with reasons | NA |
| Sample size | 7a | How sample size was determined | 3 |
|  | 7b | When applicable, explanation of any interim analyses and stopping guidelines | NA |
| Randomisation: |  |  |  |
| Sequence generation | 8a | Method used to generate the random allocation sequence | 3 |
|  | 8b | Type of randomisation; details of any restriction (such as blocking and block size) | 3 |
| Allocation concealment mechanism | 9 | Mechanism used to implement the random allocation sequence (such as sequentially numbered containers), describing any steps taken to conceal the sequence until interventions were assigned | 3 |
| Implementation | 10 | Who generated the random allocation sequence, who enrolled participants, and who assigned participants to interventions | Study protocol (Swinkels et al. 2020) |
| Blinding | 11a | If done, who was blinded after assignment to interventions (for example, participants, care providers, those assessing outcomes) and how | NA |
|  | 11b | If relevant, description of the similarity of interventions | NA |
| Statistical methods | 12a | Statistical methods used to compare groups for primary and secondary outcomes | 6 |
|  | 12b | Methods for additional analyses, such as subgroup analyses and adjusted analyses | 6 |
| **Results** | | | |
| Participant flow (a diagram is strongly recommended) | 13a | For each group, the numbers of participants who were randomly assigned, received intended treatment, and were analysed for the primary outcome | Figure 1 |
|  | 13b | For each group, losses and exclusions after randomisation, together with reasons | Figure 1 |
| Recruitment | 14a | Dates defining the periods of recruitment and follow-up | 3 |
|  | 14b | Why the trial ended or was stopped | NA |
| Baseline data | 15 | A table showing baseline demographic and clinical characteristics for each group | Table 1 |
| Numbers analysed | 16 | For each group, number of participants (denominator) included in each analysis and whether the analysis was by original assigned groups | Table 2 |
| Outcomes and estimation | 17a | For each primary and secondary outcome, results for each group, and the estimated effect size and its precision (such as 95% confidence interval) | Table 2 |
|  | 17b | For binary outcomes, presentation of both absolute and relative effect sizes is recommended | Table 2 |
| Ancillary analyses | 18 | Results of any other analyses performed, including subgroup analyses and adjusted analyses, distinguishing pre-specified from exploratory | 9, Table 3 |
| Harms | 19 | All important harms or unintended effects in each group (for specific guidance see CONSORT for harms) | NA (negative effects are discussed in results section) |
| **Discussion** | | | |
| Limitations | 20 | Trial limitations, addressing sources of potential bias, imprecision, and, if relevant, multiplicity of analyses | 14 |
| Generalisability | 21 | Generalisability (external validity, applicability) of the trial findings | 14 |
| Interpretation | 22 | Interpretation consistent with results, balancing benefits and harms, and considering other relevant evidence | 13 |
| **Other information** | | |  |
| Registration | 23 | Registration number and name of trial registry | 1 |
| Protocol | 24 | Where the full trial protocol can be accessed, if available | 3 |
| Funding | 25 | Sources of funding and other support (such as supply of drugs), role of funders | 15 |

*We strongly recommend reading this statement in conjunction with the CONSORT 2010 Explanation and Elaboration for important clarifications on all the items. If relevant, we also recommend reading CONSORT extensions for cluster randomised trials, non-inferiority and equivalence trials, non-pharmacological treatments, herbal interventions, and pragmatic trials. Additional extensions are forthcoming: for those and for up to date references relevant to this checklist, see [www.consort-statement.org](http://www.consort-statement.org).

# Supplementary Tables

## Table 1. Overview of outcome variables, instruments, and assessment timepoints from baseline assessment to 18-month follow-up

| **Outcome variables** | **Instrument, measurement type** | **Baseline** | **3 months** | **6 months** | **9 months** | **12 months** | **18 months** |
| --- | --- | --- | --- | --- | --- | --- | --- |
| **Primary outcomes** |  |  |  |  |  |  |  |
| Mental wellbeing | MHC-SF, self-report | • | • | • | • | • | • |
| **Key secondary outcomes** |  |  |  |  |  |  |  |
| Psychiatric functioning |  |  |  |  |  |  |  |
| General psychiatric functioning | HoNOS, observer-rated | • | • | • | • | • | • |
| Hospitalization | Single questions, self-report |  | • | • | • | • | • |
|  | Medical record |  |  |  |  | • | • |
| Criminal recidivism |  |  |  |  |  |  |  |
| Criminal behavior | SRD, self-report | • |  |  |  | • | • |
| Incarceration | Single questions, self-report |  | • | • | • | • | • |
| **Other secondary outcomes** |  |  |  |  |  |  |  |
| Social network |  |  |  |  |  |  |  |
| Core social network | NGI-method, self-report | • |  |  |  | • | • |
| Positive social support | SSL-I, self-report | • |  |  |  | • | • |
| Loneliness | Loneliness Scale, self-report | • |  |  |  | • | • |
| Substance use | MATE 2.1, self-report | • | • | • | • | • | • |
| Quality of life | MANSA, self-report | • | • | • | • | • | • |
| Self-sufficiency | SSM-D, observer-rated | • | • | • | • | • | • |

## Table 2. Crude and adjusted treatment effects on primary and secondary outcomes from intention-to-treat analyses (*N* = 102)

|  |  | **Descriptive statistics of raw data** | | **Between-group effects** | |
| --- | --- | --- | --- | --- | --- |
| **Outcome variables (measurement type)** | **Assessment time** | **TAU+FNC group** | **TAU group** | **Crude effect** | **Adjusted effect** |
| **Primary outcome** |  |  |  |  |  |
| Mental wellbeing (self-report)^a^ |  |  |  | -0.222 (-0.468 to 0.023)^d^ | -0.193 (-0.434 to 0.047)^d^ |
|  | Baseline | 3.797 (0.967), *n =* 50 | 3.622 (1.146), *n* = 51 |  |  |
|  | 3 months | 3.732 (0.921), *n* = 35 | 3.825 (1.083), *n* = 44 | -0.176 (-0.524 to 0.171) | -0.154 (-0.497 to 0.190) |
|  | 6 months | 3.879 (1.029), *n* = 44 | 4.054 (1.211), *n* = 39 | -0.362 (-0.701 to -0.022)* | -0.337 (-0.674 to 0.000)* |
|  | 9 months | 3.951 (0.827), *n* = 41 | 4.002 (1.092), *n* = 39 | -0.184 (-0.528 to 0.160) | -0.162 (-0.502 to 0.177) |
|  | 12 months | 3.993 (0.926), *n* = 42 | 4.033 (1.196), *n* = 42 | -0.178 (-0.515 to 0.158) | -0.151 (-0.484 to 0.182) |
|  | 18 months | 3.953 (0.918), *n* = 39 | 3.948 (1.062), *n* = 39 | -0.271 (-0.619 to 0.076) | -0.234 (-0.578 to 0.111) |
| **Key secondary outcomes** |  |  |  |  |  |
| Psychiatric functioning |  |  |  |  |  |
| General psychiatric functioning  (observer-rated)^a^ |  |  |  | 0.026 (-0.097 to 0.149)^d^ | -0,009 (-0.139 to 0.120)^d^ |
|  | Baseline | 0.866 (0.434), *n* = 51 | 0.891 (0.445), *n* = 51 |  |  |
|  | 3 months | 0.933 (0.493), *n* = 35 | 0.780 (0.365), *n* = 44 | 0.128 (-0.045 to 0.301) | 0.091 (-0.086 to 0.269) |
|  | 6 months | 0.790 (0.441), *n* = 44 | 0.737 (0.358), *n* = 39 | 0.038 (-0.132 to 0.207) | 0.003 (-0.172 to 0.177) |
|  | 9 months | 0.833 (0.469), *n* = 41 | 0.744 (0.402), *n* = 39 | 0.056 (-0.116 to 0.228) | 0.024 (-0.152 to 0.200) |
|  | 12 months | 0.772 (0.450), *n* = 42 | 0.764 (0.506), *n* = 42 | -0.034 (-0.203 to 0.135) | -0.070 (-0.243 to 0.103) |
|  | 18 months | 0.757 (0.457), *n* = 37 | 0.785 (0.487), *n* = 38 | -0.032 (-0.207 to 0.144) | -0.067 (-0.246 to 0.113) |
| Hospitalization |  |  |  |  |  |
| Number in addiction and psychiatric  care (self-report)^b^ |  |  |  | 0.936 (0.454 to 1.887)^d^ | 0.976 (0.488 to 1.952)^d^ |
|  | 3 months | 0.04 (0.208), *n* = 45 | 0.10 (0.303), *n* = 50 | 0.467 (0.100 to 2.176) | 0.450 (0.094 to 2.143) |
|  | 6 months | 0.11 (0.383), *n* = 45 | 0.09 (0.291), *n* = 44 | 1.255 (0.313 to 5.022) | 1.232 (0.308 to 4.931) |
|  | 9 months | 0.12 (0.328), *n* = 42 | 0.14 (0.516), *n* = 43 | 0.828 (0.215 to 3.189) | 0.848 (0.206 to 3.481) |
|  | 12 months | 0.07 (0.261), *n* = 42 | 0.12 (0.400), *n* = 41 | 0.584 (0.129 to 2.637) | 0.593 (0.133 to 2.647) |
|  | 18 months | 0.22 (0.584), *n* = 37 | 0.15 (0.362), *n* = 40 | 1.544 (0.512 to 4.658) | 1.635 (0.558 to 4.790) |
| Number in internal mental healthcare  institute (medical record)^b^ |  |  |  |  |  |
|  | 12 months | 0.167 (0.476), *n* = 48 | 0.192 (0.495), *n* = 47 | 0.870 (0.310 to 2.447) | 1.020 (0.304 to 3.424) |
|  | 18 months | 0.271 (0.644), *n* = 48 | 0.234 (0.520), *n* = 47 | 1.157 (0.471 to 2.841) | 1.016 (0.357 to 2.890) |
| Days in internal mental healthcare  institute (medical record)^b^ |  |  |  |  |  |
|  | 12 months | 3.354 (9.486), *n* = 48 | 24.319 (78.536), *n* = 47 | 0.138 (0.089 to 0.213)*** | 0.483 (0.252 to 0.926)* |
|  | 18 months | 5.000 (13.358), *n* = 48 | 33.851 (107.420), *n* = 47 | 0.148 (0.097 to 0.226)*** | 0.244 (0.130 to 0.455)*** |
| Criminal recidivism |  |  |  |  |  |
| Criminal behavior (self-report)^b^ |  |  |  | 0.327 (0.123 to 0.864)^d^* | 0.346 (0.152 to 0.787)^d^* |
|  | Baseline | 47.765 (103.125), *n* = 51 | 13.286 (46.142), *n* = 49 |  |  |
|  | 12 months | 26.738 (55.231), *n* = 42 | 28.275 (79.831), *n* = 40 | 0.402 (0.112 to 1.444) | 0.575 (0.225 to 1.470) |
|  | 18 months | 19.054 (50.862), *n* = 37 | 28.622 (59.668), *n* = 37 | 0.177 (0.062 to 0.508)*** | 0.180 (0.053 to 0.611)** |
|  |  |  |  |  |  |
| Incarceration (self-report)^b^ |  |  |  | 0.401 (0.159 to 1.015)^d^* | 0.451 (0.172 to 1.183)^d^ |
|  | 3 months | 0.02 (0.149), *n* = 45 | 0.14 (0.351), *n* = 50 | 0.164 (0.022 to 1.202) | 0.188 (0.023 to 1.563) |
|  | 6 months | 0.07 (0.252), *n* = 45 | 0.05 (0.309), *n* = 42 | 1.249 (0.172 to 9.095) | 1.417 (0.187 to 10.757) |
|  | 9 months | 0.07 (0.342), *n* = 42 | 0.12 (0.395), *n* = 42 | 0.544 (0.098 to 3.022) | 0.593 (0.105 to 3.342) |
|  | 12 months | 0.07 (0.261), *n* = 42 | 0.12 (0.510), *n* = 41 | 0.601 (0.114 to 3.179) | 0.670 (0.136 to 3.292) |
|  | 18 months | 0.00 (0.000), *n* = 37 | 0.15 (0.366), *n* = 39 | NA | NA |
| **Other secondary outcomes** |  |  |  |  |  |
| Social network (self-report) |  |  |  |  |  |
| Core social network |  |  |  |  |  |
| Size^a^ |  |  |  | 0.365 (-0.282 to 1.011)^d^ | 0.377 (-0.299 to 1.053)^d^ |
|  | Baseline | 3.941 (2.204), *n* = 51 | 4.078 (3.149), *n* = 51 |  |  |
|  | 12 months | 5.210 (2.928), *n* = 41 | 3.825 (2.561), *n* = 40 | 0.726 (-0.040 to 1.492) | 0.741 (-0.052 to 1.535) |
|  | 18 months | 4.324 (2.583), *n* = 37 | 4.158 (2.400), *n* = 38 | -0.051 (-0.843 to 0.741) | -0.018 (-0.831 to 0.794) |
| Quality^c^ |  |  |  | 1.042 (0.505 to 2.153)^d^ | 1.061 (0.476 to 2.362)^d^ |
|  | Baseline | 21 (41.2%), *n* = 51 | 23 (46.0%), *n* = 50 |  |  |
|  | 12 months | 19 (46,3%), *n* = 41 | 21 (52.5%), *n* = 40 | 1.307 (0.525 to 3.254) | 1.326 (0.497 to 3.539) |
|  | 18 months | 20 (54.1%), *n* = 37 | 19 (50.0%), *n* = 38 | 0.809 (0.319 to 2.054) | 0.834 (0.310 to 2.240) |
| Positive social support^a^ |  |  |  | 0.072 (-0.103 to 0.248)^d^ | 0.130 (-0.042 to 0.302)^d^ |
|  | Baseline | 2.163 (0.521), *n* = 51 | 2.090 (0.563), *n* = 51 |  |  |
|  | 12 months | 2.235 (0.530), *n* = 42 | 2.080 (0.557), *n* = 41 | 0.082 (-0.113 to 0.277) | 0.138 (-0.054 to 0.330) |
|  | 18 months | 2.243 (0.512), *n* = 37 | 2.110 (0.584), *n* = 39 | 0.063 (-0.138 to 0.263) | 0.121 (-0.076 to 0.318) |
| Loneliness^a^ |  |  |  | -0.054 (-0.325 to 0.216)^d^ | -0.078 (-0.349 to 0.192)^d^ |
|  | Baseline | 3.218 (0.803), *n* = 51 | 3.410 (0.769), *n* = 51 |  |  |
|  | 12 months | 2.794 (0.774), *n* = 42 | 3.031 (0.978), *n* = 41 | -0.141 (-0.454 to 0.173) | -0.162 (-0.478 to 0.153) |
|  | 18 months | 2.786 (0.831), *n* = 37 | 2.907 (0.862), *n* = 39 | 0.041 (-0.284 to 0.366) | 0.013 (-0.311 to 0.338) |
| Substance use (self-report) |  |  |  |  |  |
| Quantity alcohol^b^ |  |  |  | 1.088 (0.691 to 1.712)^d^ | 1.078 (0.682 to 1.702)^d^ |
|  | Baseline | 9.588 (14.054), *n* = 51 | 6.902 (12.911), *n* = 51 |  |  |
|  | 3 months | 8.800 (18.316), *n* = 35 | 6.682 (11.597), *n* = 44 | 0.947 (0.435 to 2.064) | 0.944 (0.428 to 2.083) |
|  | 6 months | 8.909 (13.322), *n* = 44 | 4.615 (7.454), *n* = 39 | 1.554 (0.834 to 2.896) | 1.523 (0.791 to 2.932) |
|  | 9 months | 9.659 (12.903), *n* = 41 | 4.615 (6.319), *n* = 39 | 1.646 (0.812 to 3.337) | 1.597 (0.798 to 3.197) |
|  | 12 months | 7.143 (9.651), *n* = 42 | 5.857 (9.527), *n* = 42 | 0.750 (0.379 to 1.487) | 0.750 (0.371 to 1.517) |
|  | 18 months | 5.684 (10.419), *n* = 38 | 4.135 (8.011), *n* = 37 | 0.831 (0.362 to 1.903) | 0.876 (0.398 to 1.930) |
| Quantity cannabis^b^ |  |  |  | 1.088 (0.666 to 1.779)^d^ | 1.175 (0.726 to 1.902)^d^ |
|  | Baseline | 0.510 (0.987), *n* = 51 | 0.510 (0.834), *n* = 51 |  |  |
|  | 3 months | 0.486 (0.702), *n* = 35 | 0.477 (1.023), *n* = 44 | 1.164 (0.594 to 2.280) | 1.311 (0.686 to 2.504) |
|  | 6 months | 0.727 (1.283), *n* = 44 | 0.436 (0.821), *n* = 39 | 1.668 (0.821 to 3.388) | 1.857 (0.935 to 3.692) |
|  | 9 months | 0.366 (0.536), *n* = 41 | 0.359 (0.628), *n* = 39 | 0.991 (0.475 to 2.069) | 1.064 (0.520 to 2.179) |
|  | 12 months | 0.405 (0.828), *n* = 42 | 0.524 (0.917), *n* = 42 | 0.653 (0.296 to 1.444) | 0.697 (0.306 to 1.585) |
|  | 18 months | 0.526 (0.862), *n* = 38 | 0.432 (0.765), *n* = 37 | 1.073 (0.519 to 2.216) | 1.136 (0.566 to 2.279) |
| Quantity hard drugs^b^ |  |  |  | 0.794 (0.228 to 2.766)^d^ | 0.885 (0.320 to 2.451)^d^ |
|  | Baseline | 0.216 (0.757), *n* = 51 | 0.118 (0.475), *n* = 51 |  |  |
|  | 3 months | 0.324 (0.912), *n* = 34 | 0.273 (1.662), *n* = 44 | 0.863 (0.136 to 5.489) | 0.999 (0.158 to 6.333) |
|  | 6 months | 0.250 (0.719), *n* = 44 | 0.026 (0.160), *n* = 39 | 6.211 (1.059 to 36.416)* | 6.159 (1.177 to 32.238)* |
|  | 9 months | 0.220 (0.475), *n* = 41 | 0.000 (0.000), *n* = 38 | NA | NA |
|  | 12 months | 0.238 (0.532), *n* = 42 | 0.643 (3.091), *n* = 42 | 0.313 (0.064 to 1.538) | 0.356 (0.103 to 1.232) |
|  | 18 months | 0.105 (0.311), *n* = 38 | 0.162 (0.602), *n* = 37 | 0.784 (0.173 to 3.554) | 0.725 (0.157 to 3.357) |
| Days alcohol^b^ |  |  |  | 1.020 (0.599 to 1.737)^d^ | 0.982 (0.600 to 1.608)^d^ |
|  | Baseline | 6.647 (9.492), *n* = 51 | 6.137 (9.633), *n* = 51 |  |  |
|  | 3 months | 7.571 (10.345), *n* = 35 | 7.364 (11.341), *n* = 44 | 0.717 (0.368 to 1.400) | 0.701 (0.365 to 1.345) |
|  | 6 months | 6.545 (9.217), *n* = 44 | 5.282 (9.671), *n* = 39 | 1.340 (0.713 to 2.516) | 1.378 (0.752 to 2.523) |
|  | 9 months | 7.244 (10.535), *n* = 41 | 6.538 (10.558), *n* = 39 | 1.038 (0.496 to 2.172) | 0.977 (0.494 to 1.933) |
|  | 12 months | 6.191 (9.253), *n* = 42 | 4.310 (7.588), *n* = 42 | 1.279 (0.597 to 2.738) | 1.213 (0.586 to 2.514) |
|  | 18 months | 6.368 (9.604), *n* = 38 | 4.028 (7.606), *n* = 36 | 1.026 (0.470 to 2.239) | 0.951 (0.452 to 2.001) |
| Days cannabis^b^ |  |  |  | 1.462 (0.782 to 2.732)^d^ | 1.199 (0.680 to 2.112)^d^ |
|  | Baseline | 9.000 (12.291), *n* = 51 | 8.765 (11.911), *n* = 51 |  |  |
|  | 3 months | 9.829 (12.743), *n* = 35 | 6.227 (10.376), *n* = 44 | 1.564 (0.729 to 3.356) | 1.298 (0.656 to 2.569) |
|  | 6 months | 9.250 (12.378), *n* = 44 | 5.846 (9.691), *n* = 39 | 1.126 (0.570 to 2.224) | 0.942 (0.485 to 1.831) |
|  | 9 months | 9.488 (12.892), *n* = 41 | 5.718 (10.364), *n* = 39 | 1.870 (0.840 to 4.160) | 1.499 (0.681 to 3.300) |
|  | 12 months | 8.857 (12.215), *n* = 42 | 5.000 (9.571), *n* = 42 | 1.875 (0.795 to 4.425) | 1.650 (0.706 to 3.859) |
|  | 18 months | 10.342 (13.358), *n* = 38 | 6.108 (11.007), *n* = 37 | 1.001 (0.409 to 2.448) | 0.741 (0.318 to 1.724) |
| Days hard drugs^b^ |  |  |  | 0.612 (0.215 to 1.744)^d^ | 0.693 (0.278 to 1.727)^d^ |
|  | Baseline | 2.078 (5.932), *n* = 51 | 0.412 (1.780), *n* = 51 |  |  |
|  | 3 months | 2.514 (6.428), *n* = 35 | 0.136 (0.554), *n* = 44 | 1.999 (0.545 to 7.331) | 2.482 (0.687 to 8.970) |
|  | 6 months | 2.295 (7.248), *n* = 44 | 0.897 (4.828), *n* = 39 | 0.277 (0.050 to 1.519) | 0.345 (0.070 to 1.778) |
|  | 9 months | 2.244 (6.822), *n* = 41 | 0.615 (2.592), *n* = 39 | 0.651 (0.161 to 2.638) | 0.931 (0.250 to 3.469) |
|  | 12 months | 0.810 (3.133), *n* = 42 | 1.071 (3.502), *n* = 42 | 0.539 (0.082 to 3.545) | 0.418 (0.077 to 2.274) |
|  | 18 months | 2.237 (6.158), *n* = 38 | 0.405 (1.423), *n* = 37 | 0.998 (0.272 to 3.659) | 1.123 (0.285 to 4.419) |
| Quality of life (self-report)^a^ |  |  |  | -0.175 (-0.504 to 0.154)^d^ | -0.147 (-0.499 to 0.204)^d^ |
|  | Baseline | 4.172 (1.106), *n* = 51 | 4.029 (0.964), *n* = 51 |  |  |
|  | 3 months | 4.212 (1.219), *n* = 35 | 4.305 (1.072), *n* = 44 | -0.090 (-0.525 to 0.345) | -0.067 (-0.520 to 0.386) |
|  | 6 months | 4.308 (1.305), *n* = 44 | 4.524 (1.119), *n* = 39 | -0.296 (-0.724 to 0.133) | -0.270 (-0.717 to 0.178) |
|  | 9 months | 4.399 (1.261), *n* = 41 | 4.500 (1.040), *n* = 39 | -0.085 (-0.518 to 0.348) | -0.085 (-0.518 to 0.348) |
|  | 12 months | 4.611 (1.195), *n* = 42 | 4.701 (1.110), *n* = 42 | -0.136 (-0.562 to 0.290) | -0.112 (-0.556 to 0.333) |
|  | 18 months | 4.432 (1.319), *n* = 39 | 4.540 (1.066), *n* = 39 | -0.344 (-0.781 to 0.092) | -0.322 (-0.776 to 0.132) |
| Self-sufficiency (observer-rated)^a^ |  |  |  | -0.001 (-0.141 to 0.138)^d^ | 0.023 (-0.124 to 0.169)^d^ |
|  | Baseline | 3.440 (0.395), *n* = 51 | 3.424 (0.346), *n* = 51 |  |  |
|  | 3 months | 3.466 (0.470), *n* = 35 | 3.517 (0.424), *n* = 44 | -0.042 (-0.224 to 0.140) | -0.020 (-0.208 to 0.168) |
|  | 6 months | 3.540 (0.535), *n* = 44 | 3.562 (0.527), *n* = 39 | -0.005 (-0.184 to 0.174) | 0.020 (-0.166 to 0.205) |
|  | 9 months | 3.580 (0.525), *n* = 41 | 3.645 (0.501), *n* = 39 | -0.045 (-0.226 to 0.136) | -0.024 (-0.210 to 0.163) |
|  | 12 months | 3.575 (0.542), *n* = 42 | 3.581 (0.504), *n* = 42 | 0.047 (-0.131 to 0.225) | 0.070 (-0.114 to 0.254) |
|  | 18 months | 3.611 (0.556), *n* = 37 | 3.623 (0.492), *n* = 38 | -0.002 (-0.186 to 0.182) | 0.019 (-0.171 to 0.209) |
| Data were analyzed with LMM/GEE/GLM and presented as adjusted mean differences (95% CI) with standardized effect sizes for continuous outcomes ^a^, rate ratios (95% CI) for count outcomes ^b^, and odds ratio (95% CI) for the dichotomous outcome ^c^. ^d^ Between-group effect on average over time. * *p*<0.05; ** *p*<0.01; *** *p*<0.001. | | | | | |
